# Preliminary Studies of the Performance of Quinoa (C*henopodium quinoa* Willd.) Genotypes under Irrigated and Rainfed Conditions of Central Malawi

**DOI:** 10.3389/fpls.2017.00227

**Published:** 2017-02-27

**Authors:** Moses F. A. Maliro, Veronica F. Guwela, Jacinta Nyaika, Kevin M. Murphy

**Affiliations:** ^1^Bunda College Campus, Lilongwe University of Agriculture and Natural ResourcesLilongwe, Malawi; ^2^Chancellor College, University of MalawiLilongwe, Malawi; ^3^Sustainable Seed Systems Lab, Department of Crop and Soil Sciences, Washington State UniversityPullman, WA, USA

**Keywords:** quinoa, variety, drought tolerance, seed yield, harvest index

## Abstract

The goal of sustainable intensification of agriculture in Malawi has led to the evaluation of innovative, regionally novel or under-utilized crop species. Quinoa (*Chenopodium quinoa* Willd.) has the potential to provide a drought tolerant, nutritious alternative to maize. We evaluated 11 diverse varieties of quinoa for their yield and agronomic performance at two locations, Bunda and Bembeke, in Malawi. The varieties originated from Ecuador, Chile and Bolivia in South America; the United States and Canada in North America; and, Denmark in Europe, and were chosen based on their variation in morphological and agronomic traits, and their potential for adaptation to the climate of Malawi. Plant height, panicle length, days to maturity, harvest index, and seed yield were recorded for each variety under irrigation at Bunda and Bembeke, and under rainfed conditions at Bunda. Plant height was significantly influenced by both genotype and environment. There were also significant differences between the two locations for panicle length whereas genotype and genotype × environment (G × E) interaction were not significantly different. Differences were found for genotype and G × E interaction for harvest index. Notably, differences for genotype, environment and G × E were found for grain yield. Seed yield was higher at Bunda (237–3019 kg/ha) than Bembeke (62–692 kg/ha) under irrigated conditions. The highest yielding genotype at Bunda was Titicaca (3019 kg/ha) whereas Multi-Hued was the highest (692 kg/ha) at Bembeke. Strong positive correlations between seed yield and (1) plant height (*r* = 0.74), (2) days to maturity (*r* = 0.76), and (3) biomass (*r* = 0.87) were found under irrigated conditions. The rainfed evaluations at Bunda revealed significant differences in seed yield, plant biomass, and seed size among the genotypes. The highest yielding genotype was Black Seeded (2050 kg/ha) followed by Multi-Hued (1603 kg/ha) and Bio-Bio (1446 kg/ha). Ecuadorian (257 kg/ha) was the lowest yielding genotype. In general the seed yields of the genotypes were lower under rainfed conditions than under irrigated conditions at Bunda. The results also highlight the need to continue evaluating a diverse number of cultivars to select for genotypes adapted to specific agro-ecological areas and across seasons in Malawi.

## Introduction

Quinoa (*Chenopodium quinoa* Willd.) is a highly nutritious Andean seed crop that has been a staple food for over 5000 years for the Inca Empire and among pre-Columbian Andean farming communities in South America (Wilson, 1990; Schlick and Bubenheim, 1996; Bhargava et al., 2007). Quinoa was domesticated in the Lake Titicaca region of Bolivia and Peru, and these countries remain the major producers and exporters of quinoa.

Quinoa has been recognized as a highly nutritious crop (Cusack, 1984; Jacobsen and Mujica, 2003; Wu et al., 2016), and demand for quinoa has soared in recent years in developed countries where there is more consciousness about wellness through healthy diets. The seed of quinoa is high in protein, possesses a balanced amino acid profile compared to common cereal grains, and is gluten free (Oelke et al., 1992; Alberta Agriculture Food and Rural Development [AAFRD], 2005; Wu et al., 2014). The increasing popularity of quinoa has triggered intensive breeding and agronomic and food science research and to promote its production and meet the growing market demand, including investigations of processing characteristics and market class opportunities (Aluwi et al., 2016; Kowalski et al., 2016).

Quinoa has the capacity to grow in a wide range of climatic conditions (Rojas et al., 2000; Garcia et al., 2003; Jacobsen et al., 2003). Quinoa cultivation has transcended continental boundaries such that it is now grown in several European countries including France, England, Sweden, Denmark, Holland, and Italy, as well as in China, India, Pakistan, New Zealand, Australia, Canada, and the United States, among many others (Jacobsen, 2003; Peterson and Murphy, 2014, 2015b; Bazile et al., 2016). According to Jacobsen and Mujica (2003), there is excellent potential for successfully growing quinoa where environmental conditions are similar to those of the Andean region. In areas where frost occurs, quinoa can survive night frost (-8°C for 2–4 h) (Jacobsen et al., 2007). Quinoa has been reported to have high salt tolerance (Adolf et al., 2012; Peterson and Murphy, 2015a; Ruiz et al., 2016) and can grow under extremely dry conditions (Sun et al., 2014; Walters et al., 2016), including drought prone areas of Africa (Jacobsen et al., 2003).

In developing countries, particularly in Africa, the introduction of high yield, domestically grown quinoa into the diet has the potential to contribute to food and nutritional security (National Statistical Office [NSO], 2010; Babatunde et al., 2011; Food and Agriculture Organization of the United Nations [FAO], 2012a,b; United Nations [UN], 2012). With the growing demand for quinoa grain in the USA, Europe, and Asia (Jacobsen and Stølen, 1993), the crop is a potential innovative and economically promising export crop for many African countries such as Malawi. The potential for the successful introduction of quinoa into African farming systems is high as the crop is adapted to a wide range of climates and ecological zones in the Andean region where it originates. Africa can therefore take advantage of the growing world demand to produce quinoa for export (Jayne et al., 2003) in addition to contributing to its own food security.

Quinoa was first introduced to Africa in the late 1990s in Kenya and Ethiopia (Oyoo et al., 2010) and more recently in Malawi. The introduction of quinoa to Malawi was prompted by the potential contribution the crop can make to improve overall sustainable intensification of the agriculture of Malawi (Maliro and Guwela, 2015). Identification of optimally adapted varieties of quinoa to different rainfed and irrigated environments in Malawi (Ministry of Natural Resources, Energy and Environment [MoNREE], 2013) would provide an opportunity for further breeding and selection, production and consumption of quinoa and similar target environments (Maliro and Guwela, 2015; Peterson et al., 2015; Murphy et al., 2016).

The objective of this study was to introduce quinoa cultivation to Malawi and evaluate diverse quinoa varieties for plant growth and grain yield performance in two contrasting environments of Malawi, Bunda and Bembeke. In addition, we tested these genotypes under both irrigated and rainfed conditions in the more favorable agronomic environment of Bunda. Our specific aims were to identify varieties with high yields and valuable agronomic characteristics in each location, and to test for genotype × environment interactions that could provide information to breeders with the goal of developing new varieties for Malawi farmers.

## Materials and Methods

**FIGURE 1:**
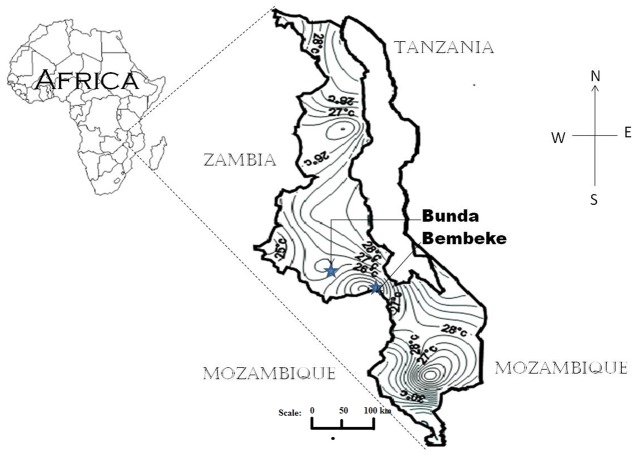
**Map of Malawi showing the locations of Bunda and Bembeke where the quinoa experiments were conducted**. The map also shows average temperature isotherms close to the locations.

Evaluation experiments were conducted at Bunda College’s Horticulture Research Farm and Bembeke Agricultural Sub Research Station. Bunda College’s Horticulture Research farm is located at 14° 12′ S and 33° 46′ E, at an elevation of 1200 m above sea level (**Figure 1**). The site receives an average rainfall of 1030 mm/year with an average temperature of 20° C. Bembeke Agricultural Sub Research Station is located at 14° 35′ S and 34° 43′ E, at an elevation of 1600 m above sea level (**Figure 1**). It receives an average annual precipitation of 1500 mm with an average temperature of 15°C (Kathabwalika et al., 2013).

Eleven varieties of quinoa were evaluated for plant growth and grain yield in two separate experiments, irrigated (two location years) and rainfed (one location year). The 11 varieties were chosen based on their variation in morphological and agronomic traits, and their potential for adaptation to the climate of Malawi. The varieties originated from Ecuador (*n* = 1), Chile (*n* = 2), and Bolivia (*n* = 1) in South America, the United States (*n* = 4) and Canada (*n* = 1) in North America, and, Denmark (*n* = 2) in Europe (**Table 1**). Evaluation for seed yield and agronomic traits under irrigated conditions was conducted in the two diverse agroecological areas of Bunda and Bembeke to better understand the potentially differing effect of temperature on the variety treatments. Evaluation of varietal treatments was conducted under rainfed conditions was done at Bunda only to get a preliminary sense of the potential for quinoa to be grown in Malawi without irrigation. The experimental design for both experiments was the same where the varieties were planted in a randomized complete block design (RCBD) with four replicates per variety. The genotypes were grown on 4 m^2^ (2 m × 2 m) raised beds with plants in 20 cm spacing between rows and 10 cm spacing between plants. The irrigated experiments were conducted from 1 July through mid-October 2012. Irrigation was provided using 10 L water-cans at 2.5 L/ m^2^ applied every 2 days from sowing to maturity at which point irrigation was withdrawn to allow plants to dry for harvesting. There was no precipitation during the entire irrigated trial period in both sites. The rainfed experiment was conducted at Bunda from 28 December 2012 to 11 April 2013 with the same spacing between rows and plants. The field was kept weed free throughout the growing season.

**Table 1:** Quinoa variety, origin and background notes of the eleven varieties grown at three locations in Malawi and described in this study.

Variety	Origin	Notes
Ecuadorian	Ecuador	
Black-seeded	Colorado, USA	Developed from cross between *Chenopodium quinoa* and *Chenopodium berlandieri*. Very tall variety (>2 m tall)
Inca Red (a.k.a. Pasankalla)	Bolivia	Member of the “Salares” ecotype of quinoa
Brightest Brilliant Rainbow	Oregon, USA	Bred by Frank Morton of Wild Garden Seeds
Bio-Bio	Chile	
Cherry Vanilla	Oregon, USA	Bred by Frank Morton of Wild Garden Seeds
Multi-Hued	British Columbia, Canada	
Red Head	Oregon, USA	Bred by Frank Morton of Wild Garden Seeds
QQ74	Chile	Heat tolerant Chilean landrace
Puno	Denmark	Bred by Sven-Erik Jacobsen
Titicaca	Denmark	Bred by Sven-Erik Jacobsen

**FIGURE 2:**
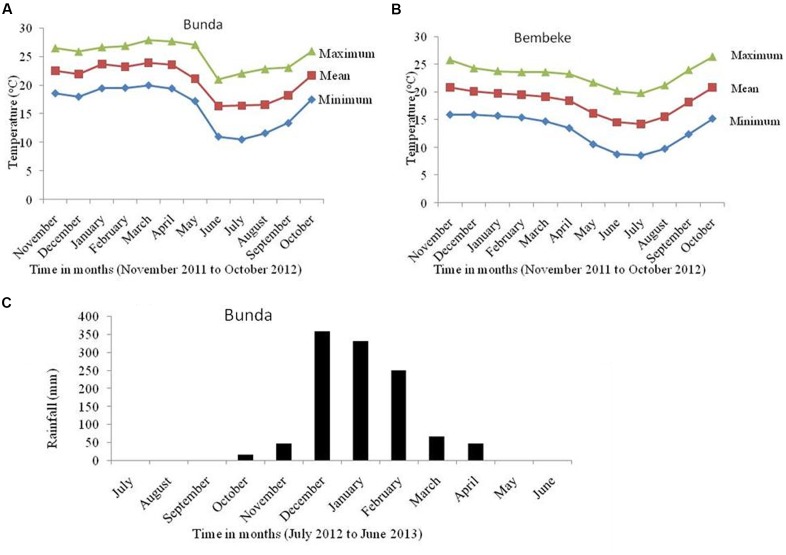
**Temperatures of Bunda (A)** and Bembeke **(B)** locations during the dry period of 2012 when the irrigated experiments were conducted and rainfall during the rainfed experiment at Bunda in 2012/2013 **(C)**.

Data was collected from a net plot which comprised an area of 2.56 m^2^ (excluding the outer 20 cm all-round the plot). Data on traits measured included number of days to maturity, plant height at harvest (cm), panicle length (cm), grain yield (kg/ha), seed weight (g/1000 seeds), and harvest index. Harvested seed and biomass per plot were weighed and yield per hectare was estimated at 12.5% moisture content. The temperatures for the two sites and rainfall for Bunda Site were recorded (**Figure 2**). The data was subjected to two-way analysis of variance where site and genotype were two factors used in the analysis using Genstat statistical package version 17. Pearson product-moment correlation (Snedecor and Cochran, 1989) analysis was used to assess the relationship of seed yield with and 1000 seed weight. Variety and location treatments were considered fixed effects and blocks and all interaction with blocks were treated as random effects.

## Results and Discussion

### Plant Height and Panicle Length

**FIGURE 3:**
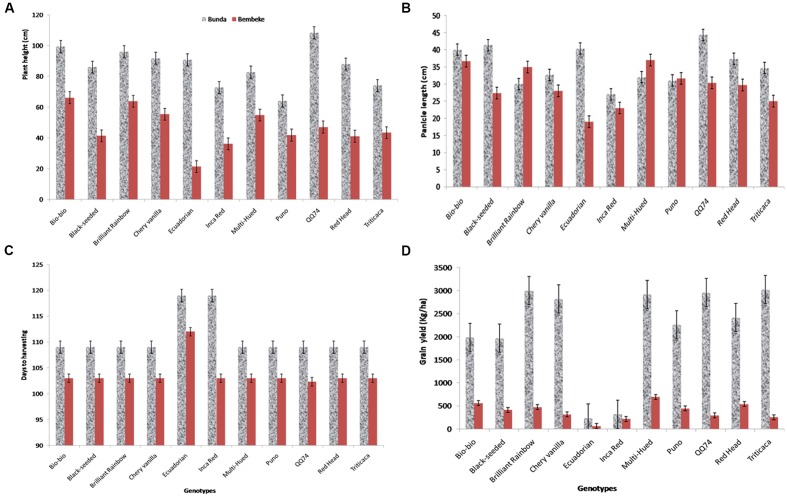
**Plant height (cm) at harvesting stage (A)** of quinoa varieties grown at Bunda and Bembeke sites (*P* = 0.002 for genotype, *P* = 0.001 for environment and *P* = 0.147 for genotype × environment, G × E), panicle length (cm) at harvesting **(B)** of quinoa varieties grown at Bunda and Bembeke sites, (*P* = 0.195 for genotype, *P* = 0.002 for environment and *P* = 0.110 for G × E), number of days taken to reach maturity **(C)** of quinoa varieties grown at Bunda and Bembeke sites (*P* = 0.001 for genotype, *P* = 0.001 for environment and *P* = 0.001 for G × E) and Grain yield (kg/ha) **(D)** of quinoa varieties grown at Bunda and Bembeke sites in Malawi from July to October 2012 (*P* = 0.001 for genotype, *P* = 0.001 for environment and *P* = 0.001 for G × E).

Under irrigated conditions, plant height at harvest was significantly influenced by both genotype (*P* ≤ 0.002) and location (*P* ≤ 0.001). The interaction between the genotype and site was not significant (**Table 2**). QQ74 grew to 108 cm tall at Bunda, followed by Bio-Bio (98 cm) (**Figure 3**). The plants were significantly shorter at Bembeke, with a mean height of 47 cm compared to 87 cm at Bunda (**Figure 3**). Bembeke is on average 5°C cooler than Bunda (**Figure 2**), and receives approximately 500 mm more precipitation per year. It is likely that the cooler temperatures were responsible for the shorter plant height, as cultivation occurred during the dry season and therefore irrigation would have ensured approximately equal access to water across both locations. Low temperatures similar to those experienced at Bembeke slow down enzymatic activity and leading to slow plant growth (Adams et al., 2001). At Bembeke, Bio-Bio grew to a height of 66 cm, followed by Brightest Brilliant Rainbow (64 cm) (**Tables 2**, **3**). Plant height was strongly correlated to seed yield (0.74), biomass (0.80), and days to maturity (0.76), but showed no association with the harvest index (**Table 2**).

**Table 2:** Performance of quinoa genotypes planted at Bunda College Horticultural Research Farm (BD) and Bembeke Sub-Research Station (BK) and grown under irrigated conditions from June to Mid October, 2012.

Varieties/lines	Plant height (cm)		Panicle length (cm)		Biomass (g/1000)		Seed size (Kg/ha^-1^)		Harvest index		Grain yield (Kg/ha^-1^)	
	BD	BK	BD	BK	BD	BK	BD	BK	BD	BK	BD	BK
Black Seeded	86.0	41.3	41.33	27.33	7434	682	0.25	0.98	0.26	0.48	1966	413
Multi-Hued	82.7	54.7	32.00	37.00	3781	2794	0.34	1.28	0.29	0.27	2916	692
Bio-Bio	99.3	66.0	40.00	36.67	7507	2752	0.19	1.01	0.27	0.20	1983	559
Brightest BR	96.0	63.7	30.00	35.00	8907	1994	0.30	1.16	0.37	0.24	2997	473
Red Head	88.0	41.0	37.33	29.67	7230	598	1.00	1.04	0.27	0.30	2413	537
Cherry Vanilla	91.7	55.3	32.67	28.00	8549	1064	1.11	1.14	0.33	0.24	2818	315
Inca Red	72.7	36.0	27.00	23.00	3034	1582	0.45	1.03	0.10	0.16	317	217
Titicaca	74.0	43.3	34.67	25.00	5580	955	0.28	1.10	0.56	0.32	3019	255
QQ74	108.3	47.0	44.33	30.33	7261	1414	0.29	1.20	0.41	0.50	2954	292
Puno	64.0	41.7	31.00	31.67	9895	2500	0.34	1.28	0.31	0.32	2260	446
Ecuadorian	90.7	21.3	40.33	19.00	2584	246	0.14	0.19	0.15	0.29	237	62

Mean	86.7	46.5	35.52	29.33	7173	1443	0.42	1.03	0.30	0.30	2171	388
LSD (0.05)	23.04		12.71		499.9		0.05		0.18		763.8	
Variety (*P*-value)	0.002		0.195		<0.001		<0.001		0.001		0.001	
Site (*P*-value)	0.001		0.002		<0.001		<0.001		0.998		0.001	
Variety × Site (*P*-value)	0.147		0.110		<0.001		<0.001		0.039		0.001	
±SE	3.89		1.92		247.3		0.026		0.034		117.9	

**Table 3:** Correlation analysis for biomass, plant height, days to maturity, seed yield and harvest index of quinoa varieties grown at Bunda and Bembeke sites in Malawi under irrigation.

	Biomass	Plant height	Days to maturity	Seed yield
Plant height	0.80^∗∗∗^			
Days to maturity	0.86^∗∗∗^	0.76^∗∗∗^		
Seed yield	0.87^∗∗∗^	0.74^∗∗∗^	0.76^∗∗∗^	
Harvest index	-0.03	0.07	-0.05	0.28

*^∗∗∗^means significant with *p* < 0.001*.

Panicle length at harvest under irrigated conditions was different across environments (*P* ≤ 0.005), but did not vary across genotype (**Table 2**). The quinoa grown at Bunda had a mean panicle length of 35.5 cm and those at Bembeke had a mean panicle length of 29 cm (**Table 2** and **Figure 3**). No genotype × environment interaction was found for panicle length. Panicle length is a yield component in quinoa and significant variation in this parameter may entail significant grain yield differences in quinoa (Long Nguyen, 2016). Genotypes with longer panicles are generally expected to give more grain yield than those with shorter panicles. In this study panicle length differences were attributed to genotype differences rather than site effect.

### Days to Maturity

Under irrigation, a significant genotype × environment interaction (*P* ≤ 0.001) was found for days to maturity. The genotypes and the site were also significantly different independent of each other at (*P* ≤ 0.001) and (*P* ≤ 0.001) respectively. Maturity was strongly correlated to plant height (0.80), plant height (0.76), and seed yield (0.76), but did not show any relationship with harvest index (**Table 3**). Jacobsen et al. (1996) also reported a positive association of quinoa grain yield with maturity period (plant cycle), plant height, length and diameter of inflorescence. This association is expected as the genotypes that were early to flower, were taller at harvest and accumulated the most biomass which translated to the highest dry grain yield per ha.

The variety Ecuadorian was the slowest to mature, taking 119 days at Bunda and 112 days at Bembeke. Inca Red (Pasankala) was also slow to mature (119 days) at Bunda (**Table 2** and **Figure 3**). These results are consistent with those of Spehar and Santos (2005), who reported that variation in days to maturity, was found to be similar among quinoa genotypes, independent of sowing dates when evaluating them across four sites in Brazil. In these results they observed that sensitivity to photoperiod and temperature in quinoa was a function of origin. Cultivars originating from the equatorial tropics were more sensitive to photoperiod and had a longer vegetative phase. Cultivars adapted to the altiplano of Peru and Bolivia were increasingly less sensitive to photoperiod the farther they originated from the equator, with the shortest vegetative phase occurring in genotypes from the highest latitudes, such as from southern Chile, northern United States, or northern Europe. This evidence indicates that to characterize growth and development of quinoa, it is necessary to analyze the response to temperature and photoperiod in all developmental phases and using a large number of genotypes (Bertero et al., 2004).

### Grain Yield

A significant interaction was found between genotype and environment (*P* ≤ 0.001) for grain yield under irrigation (**Table 2**). Both genotype and environment effects were significantly different (*P* ≤ 0.001). Higher yields were obtained at Bunda (237–3019 kg/ha; **Table 2**) compared to Bembeke (62–692 kg/ha) (**Figure 3**). This could be the result of lower temperatures at Bembeke compared to Bunda during the time the experiments were conducted (**Figure 2**). Temperature is the main abiotic factors affecting quinoa growth, germination and productivity. Hirich et al. (2014) showed that low temperatures negatively affected the productivity of quinoa by increasing length of growing period. According to research conducted by Jacobsen and Bach (1998), the optimal temperature of growth for quinoa is approximately 22°C, and Bunda was closer to this ideal than Bembeke.

The highest yielding variety at Bunda was Titicaca (3019 kg/ha), exhibiting exceptional yield potential for optimizing sustainable intensification strategies in mid-elevation locations where irrigation is available. Multi-Hued was the highest yielding variety (692 kg/ha) at Bembeke (**Figure 3**). Multi-Hued was developed in Canada, where cooler temperatures are common during the growing season, therefore it is not surprising this variety performed well in the lower temperatures found at Bembeke. The lowest yielding variety at both sites was Ecuadorian which yielded 237 kg/ha at Bunda and 62 kg/ha at Bembeke (**Table 2** and **Figure 3**). Ecuadorian also was slowest to reach maturity at both sites (112–119 days) as compared to the mean of the rest of the genotypes (101–103 days). This delayed maturity is likely a primary reason for the low yields shown in Ecuadorian. Seed yield was strongly correlated to biomass (*r* = 0.87; *p* < 0.001), plant height (*r* = 0.74; *p* < 0.001) and days to maturity (*r* = 0.76; *p* < 0.001) under irrigation. The faster maturing varieties were typically among the highest yielding. Yield differences among quinoa varieties are commonly observed, often due to differences in heat or drought tolerance (Pulvento et al., 2010; Gonzalez et al., 2012). For example, a 2 years study in Italy reported that Regalona Baer performed better than KVLQ520Y under rainfed conditions, most likely due to higher tolerance to high temperatures associated with water stress (Pulvento et al., 2010).

When grown under rainfed conditions at Bunda, differences (*P* ≤ 0.001) were found for grain yield, plant biomass and seed size among the 11 varieties (**Table 4**). The highest yielding genotype was Black Seeded (2050 kg/ha) followed by Multi-Hued (1603 kg/ha) and Bio-Bio (1446 kg/ha). Similar results of these two varieties were obtained from the trials under irrigated conditions where Multi-Hued was the highest performer in Bembeke (624 kg/ha) and fourth highest performer in Bunda (2916 kg/ha). This indicates potential for broad adaptation in the variety Multi-Hued, whereas others are perhaps more narrowly adapted to specific environments. For example, Bio-Bio and Black Seeded were the 2nd and 4th highest performers in Bembeke, but among the lowest performers in Bunda. Further testing in multiple years across multiple locations in Malawi is needed to better understand the adaptation potential in these varieties. In general, varieties grown at Bunda under rainfed conditions were lower yielding compared to the same varieties at the same location under irrigated conditions. This was due in part to the poor establishment of the crop in the rainy season, as some plants were heavily affected by heavy storm damage post-emergence. These results support other studies that show mitigation of plant stresses may help to increase yields. Geerts et al. (2008) found that well-planned deficit irrigation can stabilize quinoa yields between 1.2 and 2 Mg/ha in the central Bolivian Altiplano. For maximum production, drought stress should be mitigated by irrigation during plant establishment, flowering and early grain fill (Geerts et al., 2008).

**Table 4:** Performance of quinoa genotypes planted at Bunda college farm and grown under rain fed conditions during 2012–2013 growing season.

Varieties/lines	Plant height	Panicle length	Biomass yield	Seed weight	Harvest index	Grain yield
	(cm)	(cm)	(Kg/ha^-1^)	(g/1000)		(Kg/ha^-1^)
Black Seeded	67.3	59.7	3163	2.1	0.29	2050
Multi-Hued	74.7	60.4	3612	2.0	0.18	1603
Bio-Bio	68.3	62.3	2924	2.0	0.19	1446
Brightest BR	73.6	40.7	2644	2.2	0.23	1386
Rosa Junin	90.3	73.8	2494	1.9	0.15	891
Red Head	69.5	56.4	2184	2.2	0.14	789
Cherry Vanilla	73.8	52.0	2018	2.4	0.15	756
Inca Red	56.8	42.6	1042	2.1	0.26	665
Titicaca	69.6	47.0	2003	2.1	0.13	653
QQ74	72.1	63.9	2052	2.6	0.11	566
Puno	65.0	41.5	1238	2.0	0.18	522
Ecuadorian	62.9	44.6	623	1.9	0.15	257

Mean	70.4	53.8	2166	2.14	0.18	965
LSD (0.05)	19.02	22.55	837.8	0.24	0.106	587
±SE	9.35	11.08	411.8	0.11	0.052	288

### Harvest Index

Multi-Hued had the highest biomass yield (3612 kg/ha) followed by Black Seeded (3163 g/ha) and Bio-Bio (2942 g/ha). Interestingly, varieties with the highest grain yield also produced a lot of biomass compared to the low yielding genotypes. This could be an important consideration when developing agronomic strategies for sustainable intensification of farming systems which include quinoa as a rotational crop. There were also significant differences among varieties for seed weight. Cherry Vanilla (2.4 g) and QQ74 (2.6 g) had biggest seeds whilst Ecuadorian (1.9 g) had the lowest seed weight. Bertero et al. (1999) found that seed size was reduced under long day and hot temperatures 21 days after anthesis. Seed size was reduced by 73% in the long day hot temperature treatments (16 h at 28°C) compared to the short day and cool-temperature treatments (10.25 h at 21°C) (Bertero et al., 1999).

**FIGURE 4:**
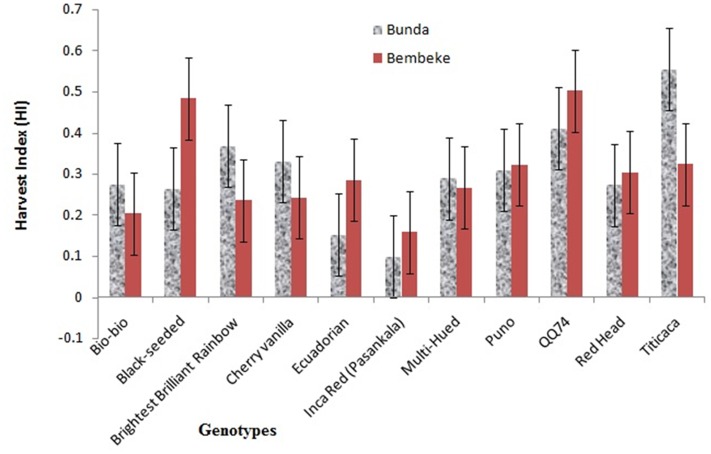
**Harvest indices (HI) of quinoa varieties grown at Bunda and Bembeke sites in Malawi from July to October 2012 (*P* = 0.001 for genotype, *P* = 0.998 for environment, and *P* = 0.039 for G × E)**.

A significant interaction between genotype and environment was found for the harvest indices (*P* ≤ 0.05). Genotype was also significantly different. Titicaca had highest harvest index (0.555) at Bunda, whereas the QQ74 had high harvest index (0.503) at Bembeke (**Figure 4**). The varieties had a harvest index range of 0.099–0.555 at Bunda and 0.203–0.503 at Bembeke. Harvest index is an excellent parameter to assess the dry matter partitioning and efficiency of plants for mobilization of photo assimilates. Most of the genotypes showed to have high efficiency in their ability to partition dry matter into photo assimilate as their harvest index ranged from 0.3 to 0.5; this resulted into high yields.

Spehar and Santos (2005) reported that positive association among dry matter production, plant height and grain yield is translated to maturity period of the quinoa plants; where later maturing genotypes grew taller than the ones that matured early, and were also shown to be superior in other yield components. However, the study results also showed exceptions for harvest index where there were low values for late and high values for early maturing genotypes, which suggest a possibility to develop quinoa for high grain and biomass production to suit different farming systems.

## Conclusion

Results of the experiments have shown that quinoa can grow well under varying agro-ecological zones in Malawi, from warmer to cooler areas. However, severely reduced grain yield may occur if quinoa is grown in the highland areas of Malawi during the winter season and emergence is insufficient. The results also highlight the need to continue evaluating a diverse number of cultivars to select for genotypes adapted to specific agro-ecological areas across seasons in Malawi. Grain yield is the basic motive for the cultivation of cereals and pseudo-cereals. Significant variation among varieties for grain yield was found at each location, indicating the importance or regional variety trials and the establishment of a quinoa breeding program in Malawi that can effectively optimize seed yield in target environments across the region.

## Author Contributions

MM conceived and conducted the field trials. JN and VG assisted in the data collection. KM provided the quinoa seed for the experiments and assisted MM in the initial development of the project. MM and KM took primary responsibility for writing the manuscript.

## Conflict of Interest Statement

The authors declare that the research was conducted in the absence of any commercial or financial relationships that could be construed as a potential conflict of interest.
